# Genetically engineered endostatin-lidamycin fusion proteins effectively inhibit tumor growth and metastasis

**DOI:** 10.1186/1471-2407-13-479

**Published:** 2013-10-15

**Authors:** Wen-guo Jiang, Xin-an Lu, Bo-yang Shang, Yan Fu, Sheng-hua Zhang, Daifu Zhou, Liang Li, Yi Li, Yongzhang Luo, Yong-su Zhen

**Affiliations:** 1 Institute of Medicinal Biotechnology, Chinese Academy of Medical Sciences and Peking Union Medical College, Beijing 100050, P. R. China; 2 Department of Pharmacology, Binzhou Medical University, Yantai, Shandong 264003, P. R. China; 3 Beijing Key Laboratory of Protein Therapeutics, Department of Biological Sciences and Biotechnology, Tsinghua University, Beijing 100084, P. R. China; 4 National Engineering Laboratory for Anti-tumor Protein Therapeutics, Tsinghua University, Beijing 100084, P. R. China

**Keywords:** Endostatin, Lidamycin, Fusion protein, Antiangiogenesis

## Abstract

**Background:**

Endostatin (ES) inhibits endothelial cell proliferation, migration, invasion, and tube formation. It also shows antiangiogenesis and antitumor activities in several animal models. Endostatin specifically targets tumor vasculature to block tumor growth. Lidamycin (LDM), which consists of an active enediyne chromophore (AE) and a non-covalently bound apo-protein (LDP), is a member of chromoprotein family of antitumor antibiotics with extremely potent cytotoxicity to cancer cells. Therefore, we reasoned that endostatin-lidamycin (ES-LDM) fusion proteins upon energizing with enediyne chromophore may obtain the combined capability targeting tumor vasculature and tumor cell by respective ES and LDM moiety.

**Methods:**

In this study, we designed and obtained two new endostatin-based fusion proteins, endostatin-LDP (ES-LDP) and LDP-endostatin (LDP-ES). In vitro, the antiangiogenic effect of fusion proteins was determined by the wound healing assay and tube formation assay and the cytotoxicity of their enediyne-energized analogs was evaluated by CCK-8 assay. Tissue microarray was used to analyze the binding affinity of LDP, ES or ES-LDP with specimens of human lung tissue and lung tumor. The in vivo efficacy of the fusion proteins was evaluated with human lung carcinoma PG-BE1 xenograft and the experimental metastasis model of 4T1-luc breast cancer.

**Results:**

ES-LDP and LDP-ES disrupted the formation of endothelial tube structures and inhibited endothelial cell migration. Evidently, ES-LDP accumulated in the tumor and suppressed tumor growth and metastasis. ES-LDP and ES show higher binding capability than LDP to lung carcinoma; in addition, ES-LDP and ES share similar binding capability. Furthermore, the enediyne-energized fusion protein ES-LDP-AE demonstrated significant efficacy against lung carcinoma xenograft in athymic mice.

**Conclusions:**

The ES-based fusion protein therapy provides some fundamental information for further drug development. Targeting both tumor vasculature and tumor cells by endostatin-based fusion proteins and their enediyne-energized analogs probably provides a promising modality in cancer therapy.

## Background

Antiangiogenesis is a promising approach to cancer therapy. As known, several antiangiogenic agents are currently under investigation in clinical trials. In contrast to those conventional therapies that kill tumor cells directly, angiogenesis inhibitors suppress tumor growth by blocking the formation of new blood vessels, which provide oxygen and nutrients for tumor growth. Endostatin (ES), a 20-kDa fragment cleaved from the collagen XVIII COOH terminus that inhibits endothelial cell proliferation and migration, is a well-known angiogenesis inhibitor, which shows antiangiogenesis and antitumor activities in several animal models. ES inhibits 65 different tumor types and modifies 12% of the human genome to down-regulate pathological angiogenesis [1]. However, the mechanism and function of ES is still insufficient understanding. For anti-angiogenic activity, ES appears to be dependent on binding to E-selectin [2]. Also, ES blocks activity of metalloproteinases 2, 9, and 13 [3]. ES may down-regulate VEGF expression in tumor cells [4]. IGF-II-mediated signaling and T-type Ca^2+^ channels also involve the function of ES [5,6].

Shi *et al*. identified that cell surface nucleolin on angiogenic blood vessels is a functional receptor for ES, and mediates the internalization and biological activities of ES [7,8]. Mechanism studies by Huang et al. show that vascular endothelial growth factor (VEGF) and extracellular matrix (ECM) synergistically induce the translocation of nucleolin from nucleus to cell surface [9-11]. Previous studies show that ES specifically binds to neovascular endothelial cells through its interaction with the integrin receptors α5β1 and αVβ3, which has been implicated in tumor metastasis [12,13]. A more recent study shows that nucleolin and integrin α5β1 can form a co-receptor for ES *via* UPAR on the endothelial cell membrane [14]. ES labeled with a near-IR probe is shown to selectively accumulate in the tumor site [15]. All these studies suggest that ES has a unique ability for targeted cancer therapy.

However, like many angiogenesis inhibitors, ES single administration didn’t achieve significant effects. The clinical development ended in the U.S. in 2003 due to limited efficacy and problems with protein formulation and application [16]. Several studies reported the improved selectivity and efficacy of chimeric molecules comprised of toxins or other cytotoxic agents with targeting agents on tumor vasculature, such as vascular endothelial growth factor receptor-gelonin; Shiga-like toxin-vascular endothelial growth factor fusion protein and anti-TES-23 linked to neocarzinostatin [17-19]. So the combination of the targeted and cytotoxic effects by engineering two independent molecules sounds to be a promising way for drug design.

Lidamycin (LDM), also called C-1027, is a member of chromoprotein family of antitumor antibiotics. The LDM molecule consists of an enediyne chromophore (AE) and a non-covalently bound apo-protein (LDP). It was shown that the AE exerts extremely potent cytotoxicity to cultured cancer cells, whereas the apo-protein LDP keeps the labile enediyne relatively stable. The non-covalently bound AE and LDP can be dissociated and re-associated. The activity of rebuilt molecule remains as potent as that of natural LDM. LDP, which is composed of 110 amino acid residues, showed specific binding capability to various human tumor tissues and displayed moderate cytotoxicity to Bel-7402 cells [20,21]. This specific binding capability and cytotoxicity of LDP implied its potential use as a targeting drug carrier in the design of new anticancer agents.

In order to combine the anti-angiogenic and cytotoxic functions of ES and LDM and to target both tumor endothelial cells and tumor cells, we designed two novel ES-based fusion proteins, ES-LDP and LDP-ES and their enediyne-energized analogs, and then detected their antitumor efficacies. Here we show that ES-LDP fusion proteins should possess targeting property of ES or LDP and moderate cytotoxicity effect of LDP in addition to antiangiogenesis activity of ES and the extremely potent cytotoxicity of the enediyne chromophore of LDM when they were assembled.

## Methods

### Cells and cell culture

HMEC cell line was maintained in endothelial-specific medium EBM-2 (Lonza, USA). The human lung carcinoma PG-BE1 was routinely grown in RPMI-1640 (HyClone,Beijing, China) supplemented with 10% fetal bovine serum (Gibco, USA), 100 U/mL penicillin, and 100 μg/mL streptomycin. The mouse breast cancer cell line 4T1 cells expressing the firefly luciferase gene (4T1-luc) were preserved in our laboratory. For stable expression, the cells were exposed to 500 μg/mL G418 (Gibco, USA). D-luciferin was purchased from Xenogen (Alameda, CA).

### Construction of the expression vectors

Two fusion proteins named LDP-ES and ES-LDP were designed with an eight-amino acid-long linker (−GGGSGGSG-) between LDP and ES. Each ES-based fusion protein gene consists of the gene encoding LDP (110 amino acids; ref. 21), ES (184 amino acids; ref. 27), and the linker peptide. After two rounds of PCR and DNA cloning process, the resultant 909-bp fragment was digested by NdeI/XhoI and was inserted into pET30a expression vector to generate the expression plasmid. DNA sequencing analysis (Invitrogen Corp.) was used to verify that the gene was correct in sequence and had been cloned in the frame.

### Wound healing assay

Cell migration was assessed in a wound-healing assay. HMEC or 4T1 Cells at 5x10^5^ cells per well were cultured in 24-wells plate provided by the CytoSelect™ 24-Well Wound Healing Assay Kit and allowed to proliferate to form a confluent monolayer. The linear spacer inserted in the well was removed, which created a regular and defined “wound” within the cell monolayer. Wash wells with media to remove dead cells and debris. Wells were treated with different concentrations of ES, ES-LDP or LDP-ES and further cultured until the control wound was fully closed at 37°C. Cells were fixed and images were captured immediately at 40X magnification from light microscopy and cells that migrated to the scraped area were counted using Image-Pro Plus 6.0 software. Each experiment was performed twice, with triplicate samples.

### Tube formation assay

Formation of capillary tube like structures by HMEC was assessed in Matrigel-based assay. Briefly, a 96-well plate coated with 60 μl of Matrigel per well was allowed to solidify at 37°C for 1 h. Cells (1.5 × 10^4^ in 100 μl medium) were added on each well and 100 μl of medium containing different concentrations of ES, ES-LDP or LDP-ES were added and incubated for different periods of time. Each treatment was performed in triplicate. The enclosed networks of tubes were photographed under microscope. The total tube lengths and numbers of the tube structure of each photograph were measured using Image-Pro Plus 6.0 software.

### Immunohistochemistry in tissue microarray

Multiple arrays of formalin-fixed, paraffin-embedded lung tumors and normal lung tissue were obtained from U.S. Biomax, Inc. (Xi’an, China). The microarray of product number BC041115a contains 110 lung tumors and unmatched normal lung tissue (10 cases/type). The normal controls were derived from the same organ but not from the same patient. The array dot diameter was 0.6 mm. All immunohistochemical studies were performed on paraffin-embedded sections as previously described [21]. For Cetuximab controls, the tissue sections were stained in the same manner except that the detection antibody was replaced with poly-HRP-anti-human IgG against Cetuximab. The positive percentage of each protein could be calculated according to the staining intensity by reference to the Herceptest™ interpretation manual.

Additionally, we analyzed the cases by Image-Pro Plus 6.0 software, using the method introduced by Xavier et al. [22,23]. Briefly, the measurement parameters included density mean, area sum, and integrated optical density (IOD). The optical density was calibrated and the area of interest was set through: hue, 0 ~ 30; saturation, 0 ~ 255; intensity, 0 ~ 255, then the image was converted to gray scale image, and the values were counted. The time required to perform the analysis process can be greatly reduced by using macro of pathology. To avoid artificial effect, cells in areas with necrosis, poor morphology, or in the margins of sections were not taken into account. The IOD were log transformed and mainly performed statistical analysis.

### Preparation of enediyne-energized ES-LDP and LDP-ES

The active enediyne chromophore (AE) of LDM was separated by using C4 column (GE Healthcare) with a 22% acetonitrile in 0.05% trifluoroactic acid mobile phase. The AE-containing solution was added to ES-LDP/PBS (10 mmol/L; pH7.4) or LDP-ES/PBS, respectively, with the molecular ratio of 4:1, and was incubated at 4°C for12 h while rocking. Free AE was removed by using a Sephadex G-75 column (GE Healthcare). Assembled enediyne-energized fusion proteins named LDP-ES-AE and ES-LDP-AE were confirmed by reverse-phase HPLC using a Vydac C4 300A column (Grace). Absorbance at 340 nm was measured.

### Cell cytotoxicity assay by cell counting kit-8

Cells were seeded at 1 × 10^4^ per well in 96-well plates and incubated in 37°C for 24 h and then exposed to different concentrations of LDM or energized fusion proteins (ES-LDP-AE, LDP-ES-AE) for 48 h. On the day of measuring the growth rate of cells, 100 μL of spent medium was replaced with an equal volume of fresh medium containing 10% CCK-8 (WST-8, Dojindo Laboratories, Tokyo, Japan). Cells were incubated at 37°C for 1 h, and cell number was assessed by measuring the absorbance at 450 nm on a microplate reader (Thermo). Three independent experiments were carried out. The IC50 represented the drug concentration resulting in 50% growth inhibition.

### Tumor models

The syngeneic murine 4T1-luc breast cancer model and human lung carcinoma PG-BE1 xenograft model have been used. The BALB/c female mice and female athymic nude mice (BALB/c, *nu*/*nu*) were purchased from the Institute for Experimental Animals, Chinese Academy of Medical Sciences & Peking Union Medical College. The study protocols were in accordance with the regulations of Good Laboratory Practice for non-clinical laboratory studies of drugs issued by the National Scientific and Technologic Committee of People’s Republic of China. The treatment and use of animals during the study was approved by the Animal Ethics Committee of the Institute of Medicinal Biotechnology, Chinese Academy of Medical Sciences & Peking Union Medical College (permission number: c1-2011-1121).

Exponentially growing human lung carcinoma PG-BE1 cells were implanted into the 16–18-week-old female athymic nude mice by the subcutaneous injection of 10 × 10^6^ cells on the right flank. After 3 weeks, the tumors were aseptically dissected and pieces of tumor tissue (2 mm^3^ in size) were transplanted s.c. separately by a trocar into athymic mice. When tumors reached about 100 mm^3^ in size, the mice were randomized into groups (n = 6 per group) and treated with ES,LDM,ES-based fusion proteins (ES-LDP, LDP-ES) and energized fusion proteins (ES-LDP-AE, LDP-ES-AE), respectively, at different doses and time intervals. Tumor growth was measured with a caliper, and tumor volumes were calculated with the following formula: V = 0.5a × b^2^, where a and b are the long and the perpendicular short diameters of the tumor, respectively. Typically, studies were terminated when tumors in the control animals reached an average size of 2000 mm^3^. Percentage of inhibition of tumor growth was calculated as 100 × {1-[(tumor volume_final_-tumor volume_initial_ for the treated group)/(tumor volume_final_-tumor volume_initial_ for the vehicle-treated group)]}.

We studied the lung metastasis of tumors using an *i*.*v*. injection model. BALB/c female mice were injected with 2 × 10^5^ murine 4T1-luc breast cancer cells in 0.2 mL PBS solution via the lateral tail vein. Three days later after tumor cell injection, mice were randomly assigned to three groups and treated with ES or ES-LDP respectively. Seven days after the first treatment, all mice were injected again at same doses. After 17 days, Mice were anesthetized with isoflurane and *i*.*p*. injected with luciferase substrate D-luciferin (150 mg/kg). The animals were placed onto the warmed stage inside the camera box (IVIS-Imaging System, Xenogen) to observe tumor growth. Then, the lungs were immediately removed, weighed and fixed in 10% buffered formalin for counting of pulmonary metastatic nodules. The metastatic nodules of 4T1 tumor in lung were counted by direct visualization using a stereomicroscope. The total number of metastases per lung section was counted and averaged among the animals.

### In vivo fluorescence imaging

When tumors reached about 200 mm^3^ in size in human PG-BE1 xenograft model, three hundred micrograms of DyLight 680-labeled ES-LDP or LDP-ES were injected *i*.*v*. (n = 3). The mice were placed under anesthesia by inhalation of isoflurane and the images were observed with the Xenogen Ivis 200 system and recorded by built-in camera (Caliper Life Sciences).

### Statistical analysis

All of the data were presented as the mean ± SD for at least three independent experiments. Statistical analysis was performed with SPSS software (version 17.0). The significant differences between any of two groups were evaluated by One-way ANOVA. Statistical significance was defined as P < 0.05.

## Results

### Construction, preparation and biochemical characterization of ES-LDP, LDP-ES and their enediyne-energized analogs

**Figure 1:**
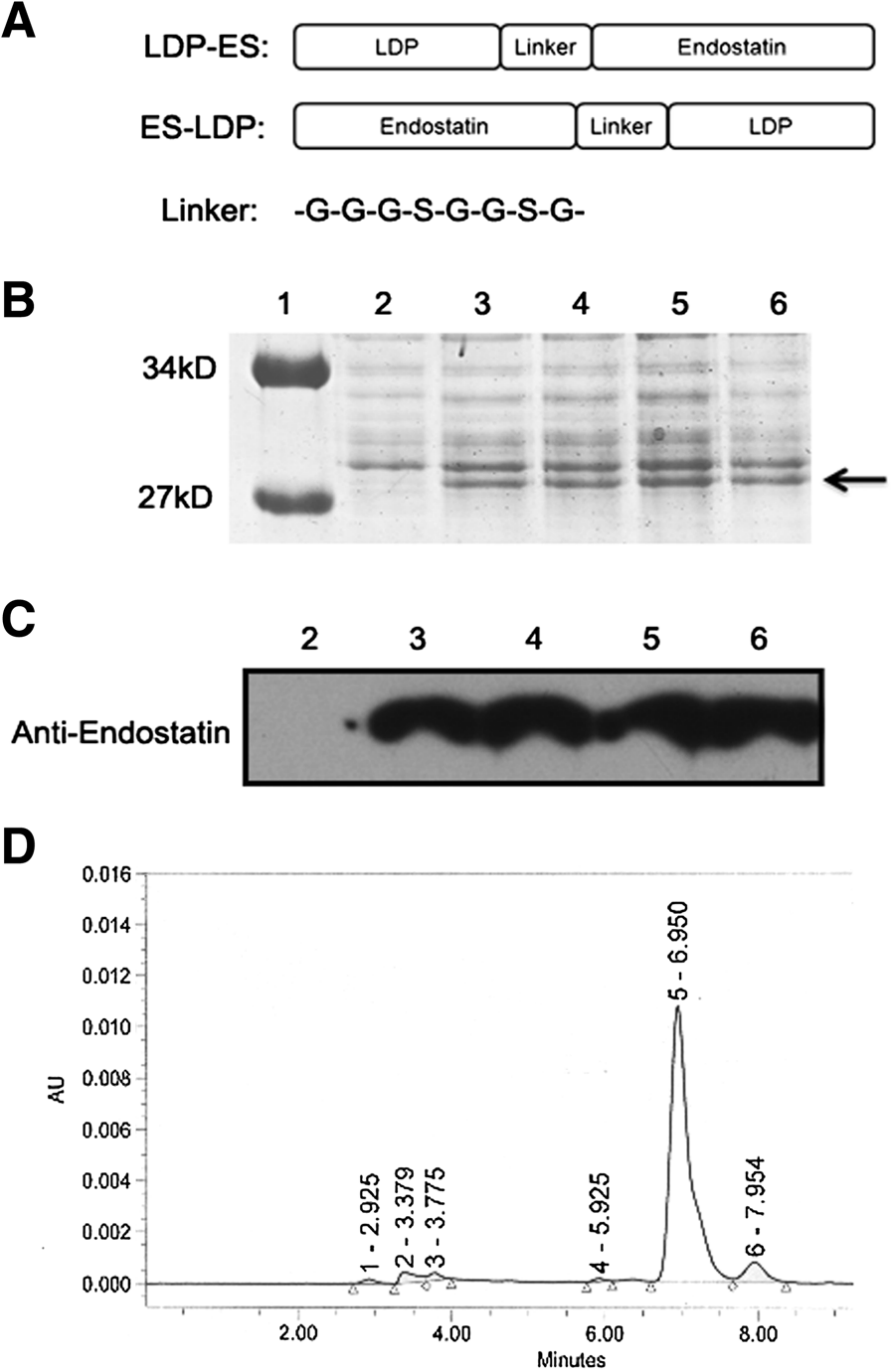
**Construction and preparation of endostatin-lidamycin fusion proteins.** Diagram of fusion proteins: LDP-ES (top) and ES-LDP (middle); Bottom, the amino acid sequence of the linker **(A)**. **(B)** SDS-PAGE and **(C)** Western blotting detection of the expression of fusion proteins. Lane 1, molecular weight marker; Lane 2, empty vector as a control; Lane 3 and Lane 4, vector expressing LDP-ES; Lane 5 and Lane 6 vector expressing ES-LDP. Arrow indicates the bands of fusion proteins. The enediyne-energized fusion proteins determined by reverse-phase HPLC on C4 300A column at 340 nm **(D)**.

DNA fragments encoding LDP and ES fusion proteins were obtained by PCR and molecular cloning techniques. As shown in Figure 1A, LDP-ES and ES-LDP were designed with an eight-amino acid-long linker between LDP and ES. The DNA fragments encoding these two fusion proteins were cloned and inserted into the pET30a expression vector. SDS-PAGE (Figure 1B) and Western blotting (Figure 1C) were used to detect the expression of fusion proteins. The energized fusion proteins were prepared by integrating AE molecule of LDM into ES-LDP and LDP-ES, respectively. Data from reverse-phase HPLC showed that AE molecule was successfully integrated into fusion proteins (Figure 1D), which implies that LDP keeps its native structure in fusion proteins. The assembling efficiency of ES-LDP and LDP-ES was 83.9% and 27.1%, respectively (data not shown).

In CCK-8 assay, LDP-ES-AE or ES-LDP-AE displayed extremely potent cytotoxicity to kinds of cancer cells and endothelial cells in proximity to that of free LDM, as shown in Table 1. The IC50 values ranged from 10^-9^ M to 10^-10^ M and all cell lines were relatively more sensitive to ES-LDP-AE than to LDP-ES-AE, which may results from the relatively lower assembling efficiency of AE in LDP-ES.

**Table 1:** **Determined IC50 values for enediyne**-**energized fusion proteins in HMEC and different cancer cell lines**

**Cells**	**IC50(M)**		
	**HMEC**	**PG**-**BE1**	**4T1**
LDM	1.16 ± 0.39 × 10^-10^	1.01 ± 0.24 × 10^-9^	2.79 ± 0.41 × 10^-10^
ES-LDP-AE	8.13 ± 0.98 × 10^-10^	8.06 ± 2.06 × 10^-10^	2.05 ± 0.67 × 10^-10^
LDP-ES-AE	1.14 ± 0.39 × 10^-9^	2.61 ± 0.14 × 10^-9^	2.94 ± 0.80 × 10^-9^

### ES-LDP and LDP-ES inhibited HMEC and 4T1 cells migration in wound healing assay

**Figure 2:**
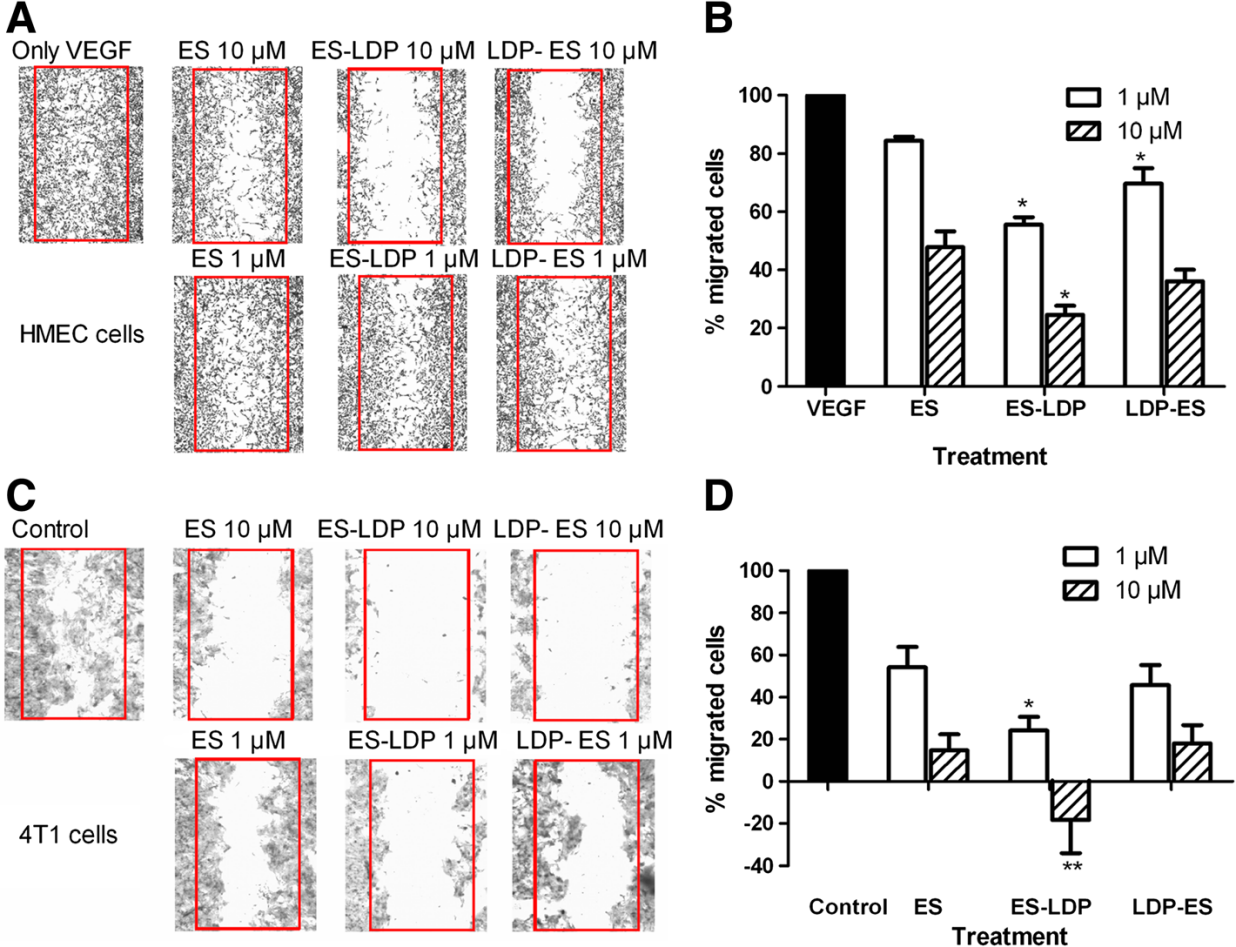
**HMEC and 4T1 migration in wound healing assay using ES or ES**-**based fusion proteins as inhibitors.** Pictures were taken at magnification 40X in light microscopy **(A** and **C)**. Quantification results of migrated cells, counted using the software Image-Pro Plus 6.0, are shown in **B** and **D**, assuming control as 100%. Inhibitors were used at indicated concentration. Results shown are average values of 6 representative fields in each of the two different experiments performed in duplicates, and error bars represent SEM. *, P ≤ 0.05, **, P ≤ 0.001, compared with ES, respectively. Cells were viewed with a microscope and pictures were taken at × 40.

New blood vessel formation requires that the endothelial cells migrate towards the sources of growth factor. We used the HMEC wound healing assay to observe the ability of ES-based fusion proteins in inhibiting endothelial cell migration. As shown in Figure 2A, cells were able to migrate towards the wound area in higher number when exogenous rhVEGF was added. ES or ES-based fusion proteins all demonstrated the ability of inhibiting HMEC migration at different concentrations when compared with rhVEGF control (Figure 2A). Comparison of quantified results shows that ES-based fusion proteins are more potent than ES, and ES-LDP exhibits a stronger inhibitory effect than LDP-ES (Figure 2B). These results indicate that ES-based fusion proteins have increased capability in inhibiting VEGF-induced endothelial cell migration.

Since 4T1 cells were reported to metastasize to the lung, liver, bone, and brain *via* the hematogenous route [24], we therefore examined effects of ES-based fusion proteins on 4T1 cell migration *in vitro* and observed similar phenomena with those in HMEC wound healing assay. As can be seen in Figure 2C and 2D, both low (1 μM) and high (10 μM) concentrations of fusion proteins markedly suppressed the migration of 4T1 cells. Interestingly, it appears that 4T1 cells are more sensitive than HMECs to ES and ES-based fusion protein treatments, and that all the proteins tested inhibited cell migration in a dose dependent manner (Figure 2).

### ES-LDP and LDP-ES disrupted endothelial tubule formation

**Figure 3:**
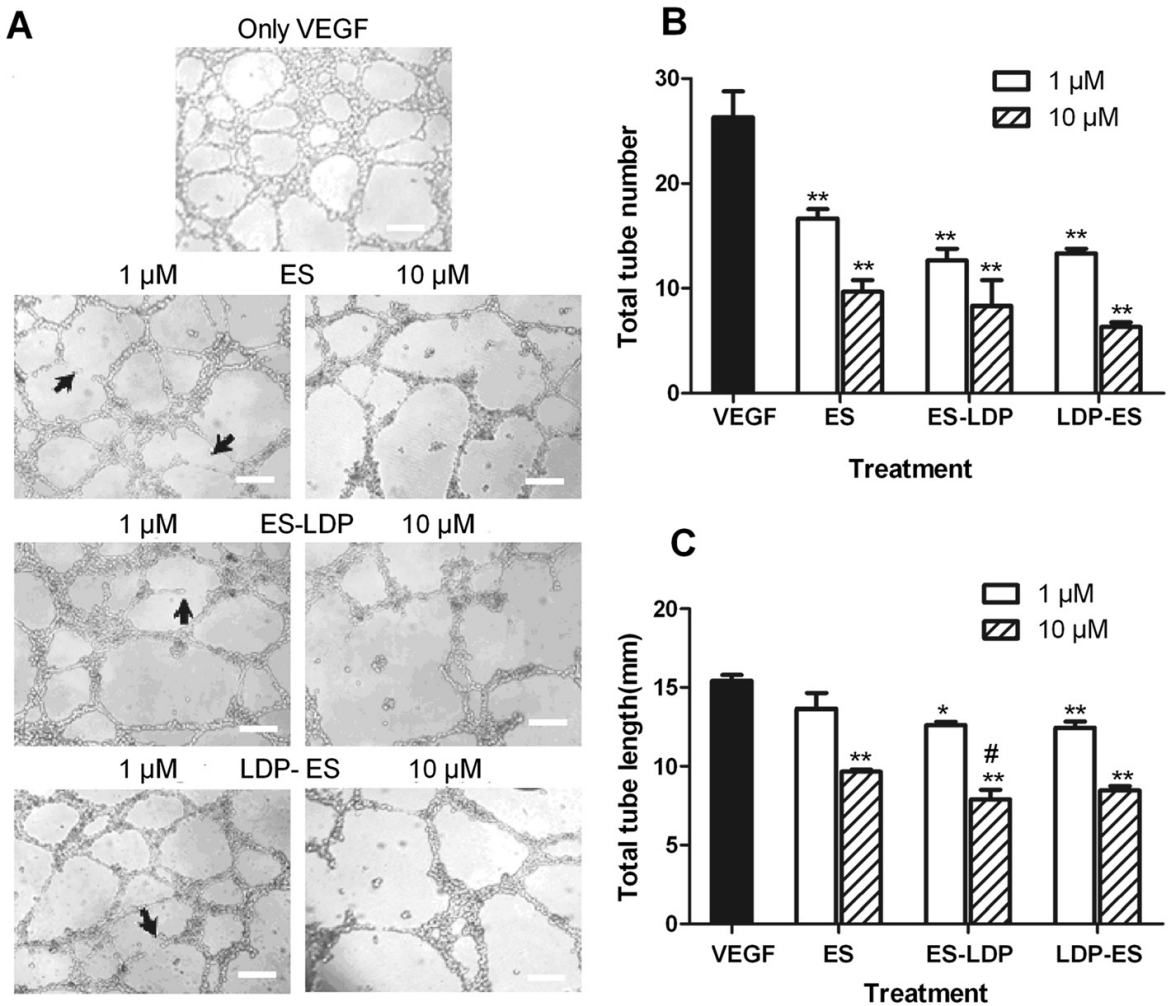
**ES**, **ES**-**LDP or LDP**-**ES inhibited in vitro tubule formation. (A)** ES or ES-based fusion proteins inhibited tubule formation of HMEC on Matrigel. Low (1 μM) and high (10 μM) concentrations were used. Bar, 200 μm. **(B)** Mean capillary tube number and **(C)** mean tube length was decreased by ES and ES-based fusion proteins after 12-h incubation. Three arbitral optical images were taken for each of the two independent experiments. Data are expressed as mean and standard deviation from six independent images, n = 6. *, P ≤ 0.05, **, P ≤ 0.001, compared with VEGF-control and #, P ≤ 0.05, compared with ES, in tube length and tube number, respectively. Cells were viewed with a microscope and pictures were taken at × 40.

An endothelial tubule formation assay was used to further confirm the antiangiogenic activity of the fusion proteins. In this experiment, the use of Matrigel permits the growth and differentiation of endothelial cells into tubal structures that are reminiscent of blood vessels. Prominent tubal structures were observed in control cells (Figure 3A). ES or ES-based fusion proteins inhibited tube formation of HMEC in a concentration-dependent manner. At the low concentration (1 μM; Figure 3A, left column) ES or ES-based fusion proteins began to disrupt the formation of the tubes, as indicated by the arrows. At the high concentration (10 μM; Figure 3A, right column), ES or ES-based fusion proteins eliminated the tubal structures. As shown in Figure 3B and 3C, ES or ES-based fusion proteins reduced the number of closed capillary tubes as well as their length.

### In vivo targeting of ES-LDP and LDP-ES

Since ES was reported to specifically target tumor tissues [15], and fusion proteins are supposed to inherit distributional specificity, *in vivo* distribution of DyLight 680-labeled ES-LDP or LDP-ES was observed in nude mice bearing human PG-BE1 xenograft.

**Figure 4:**
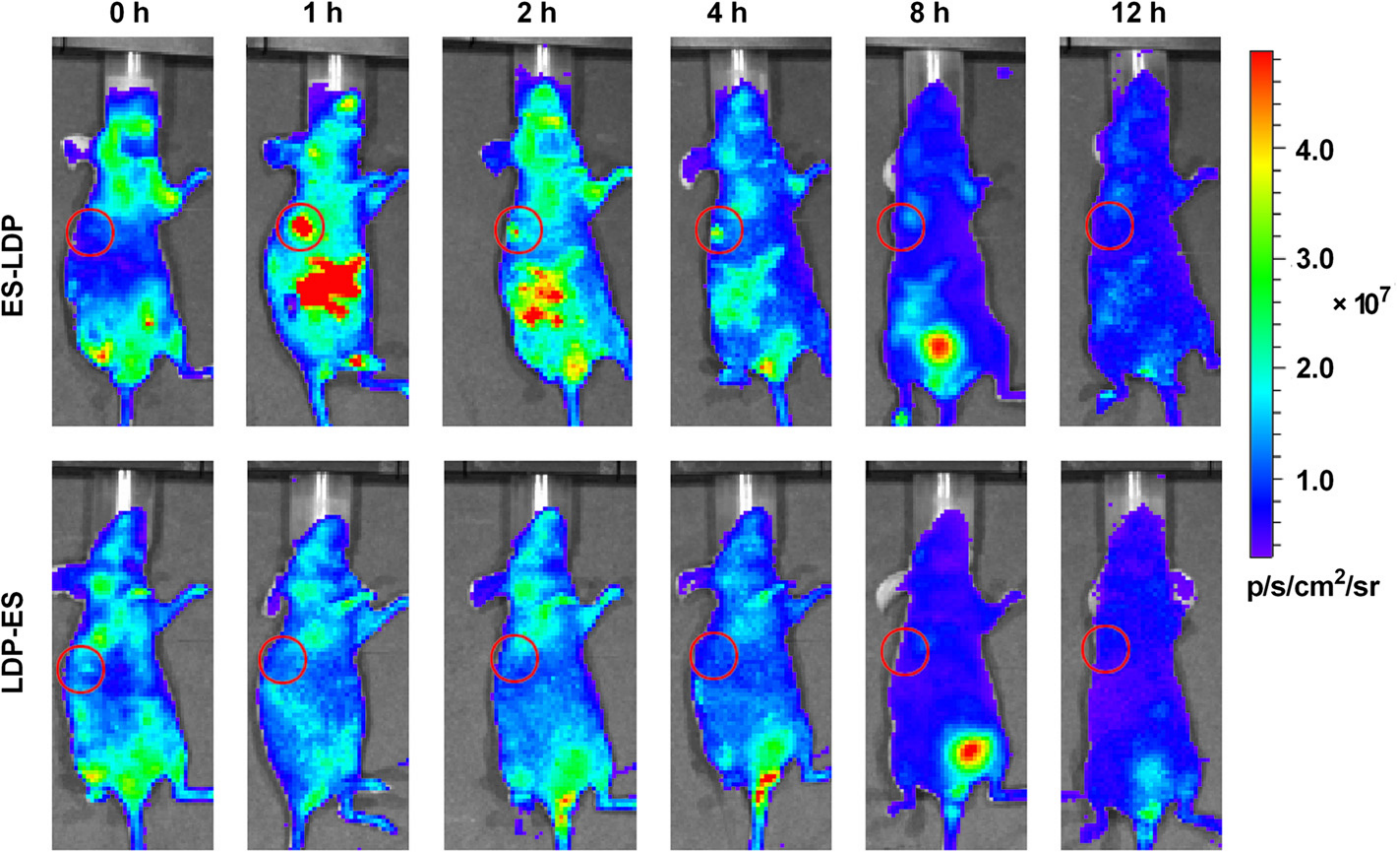
**Optical imaging in living animal using DyLight 680**-**labeled ES**-**LDP or LDP**-**ES.** Color scale represents photons/s/cm^2^/steradian, the red dot-cycle indicated the tumor location.

As expected, ES-LDP protein accumulated into the tumor area and reached the highest level within 1 h after injection and then gradually cleared from the tumor area during the following 3 hours (Figure 4, Upper). Surprisingly, DyLight 680-labeled LDP-ES showed little accumulation in PG-BE1 tumor, but a random distribution in the whole body followed by a normal clearance process (Figure 4, Lower). However, this observation is consistent with our previous result obtained with LDP [25], which indicates that fusion LDP to the N-terminus of ES does not improve the targeting of LDP.

### Binding of ES-LDP to lung tumors and normal lung tissues

**Figure 5:**
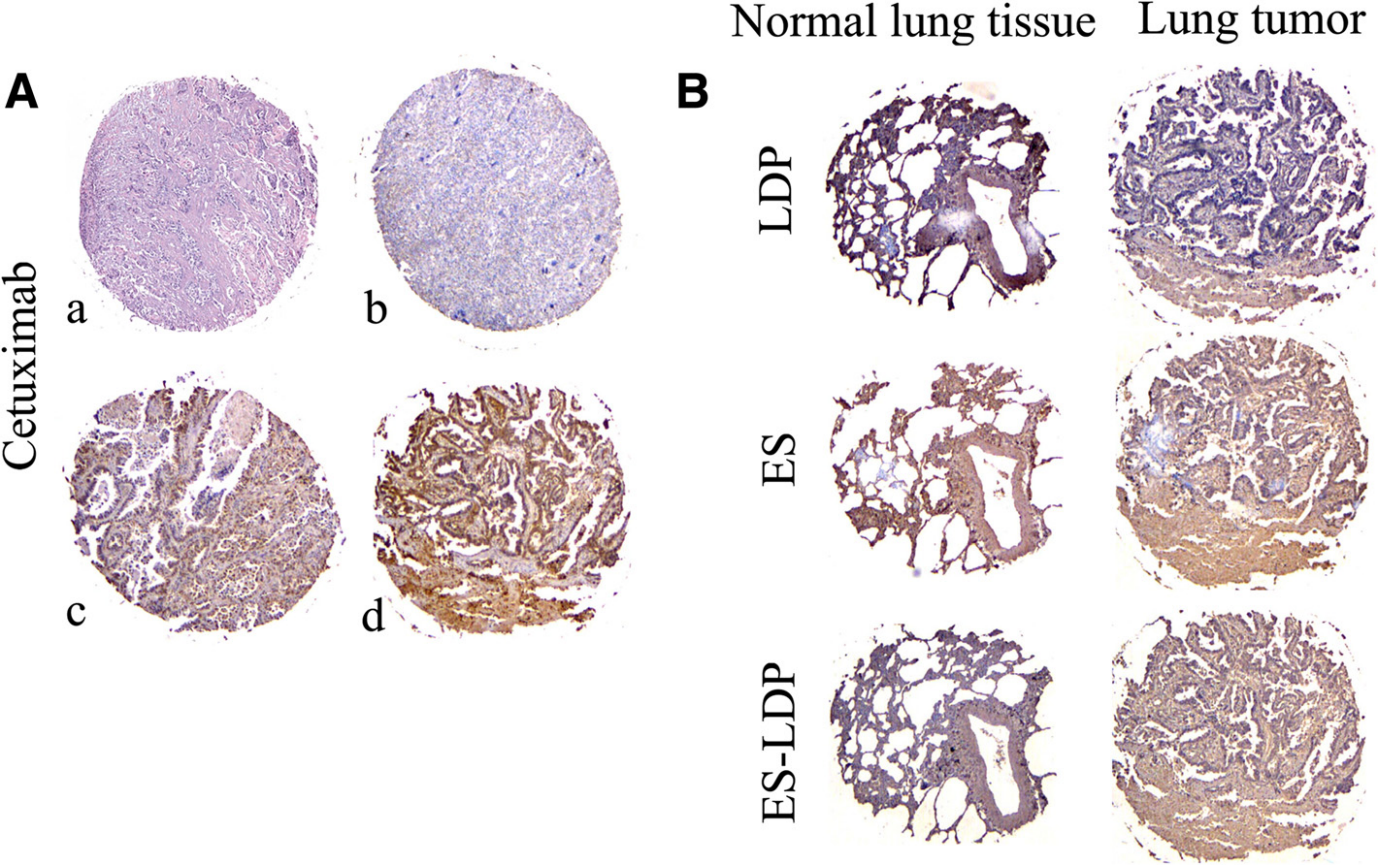
**Lung tissue microarray. (A)** Representative examples of HE staining (a); no staining (b), weak staining (c) and strong staining (d) for Cetuximab; **(B)** Representative examples of LDP, ES or ES-LDP staining. First row: normal human lung tissues with ES positive staining. Second row: lung tumor tissues with ES and ES-LDP positive. Original magnification was 40 × .

Based on the ES-LDP accumulation in human lung carcinoma PG-BE1, we tested the binding capability of LDP, ES and ES-LDP through tissue microarray of human lung tissues. The number of spots that can be interpreted was 117 from total 120 core samples (3 normal tissue cores were missed). Cetuximab was used as a control to ensure criterion of tissue microarray (Figure 5A). The representative examples of LDP, ES or ES-LDP staining were shown in Figure 5B. The positive percentage of ES and ES-LDP was higher than that of LDP (P < 0.001, χ^2^ test; Table 2). The difference of ES-LDP binding capability between the tumor tissue and normal tissue samples was significant (P < 0.05, χ^2^ test; Table 2). Additionally, the IOD value of Image Pro-Plus analysis was representative parameter to assess the immunohistochemistry quantification, and increased sensitivity in scoring and provided a more reliable and reproducible analysis of protein expression and binding capability. We compared the differences of IOD, respectively. The differences of IOD were significant between LDP and ES, as well as between LDP and ES-LDP (P < 0.0001). However, the difference between ES and ES-LDP was not significant (P > 0.05, Table 2).

**Table 2:** Summary analysis and comparison of proteins binding capability in lung tissue microarray

**Groups**	**Lung tumors (n = 107)**		**Normal lung tissues (n = 10)**	
	**Positive percentage**	**Log**_**10 **_**IOD**	**Positive percentage**	**Log**_**10 **_**IOD**
		**(Mean ± SD)**		**(Mean ± SD)**
LDP	44.9	6.59 ± 0.31	50	6.33 ± 0.28
ES	94.4^##^	7.21 ± 0.23**	90	6.59 ± 0.19
ES-LDP	87.5^##^	7.03 ± 0.28**	60^	6.39 ± 0.18

** *P* ≤ 0.001, *vs* LDP (One-way ANOVA test).

^##^*P* ≤ 0.001, *vs* LDP (χ^2^ test).

^ *P* ≤ 0.05, *vs* lung tumor (χ^2^ test).

### ES-LDP, LDP-ES and their enediyne-energized analogs inhibited tumor growth

Since ES-LDP and LDP-ES show dramatic difference in tumor targeting *in vivo*, we are thus curious about their antitumor efficacies. The *in vivo* efficacies of ES-based fusion proteins and their enediyne-energized forms were tested by two separate experiments with human lung carcinoma PG-BE1 xenograft in athymic mice.

**Figure 6:**
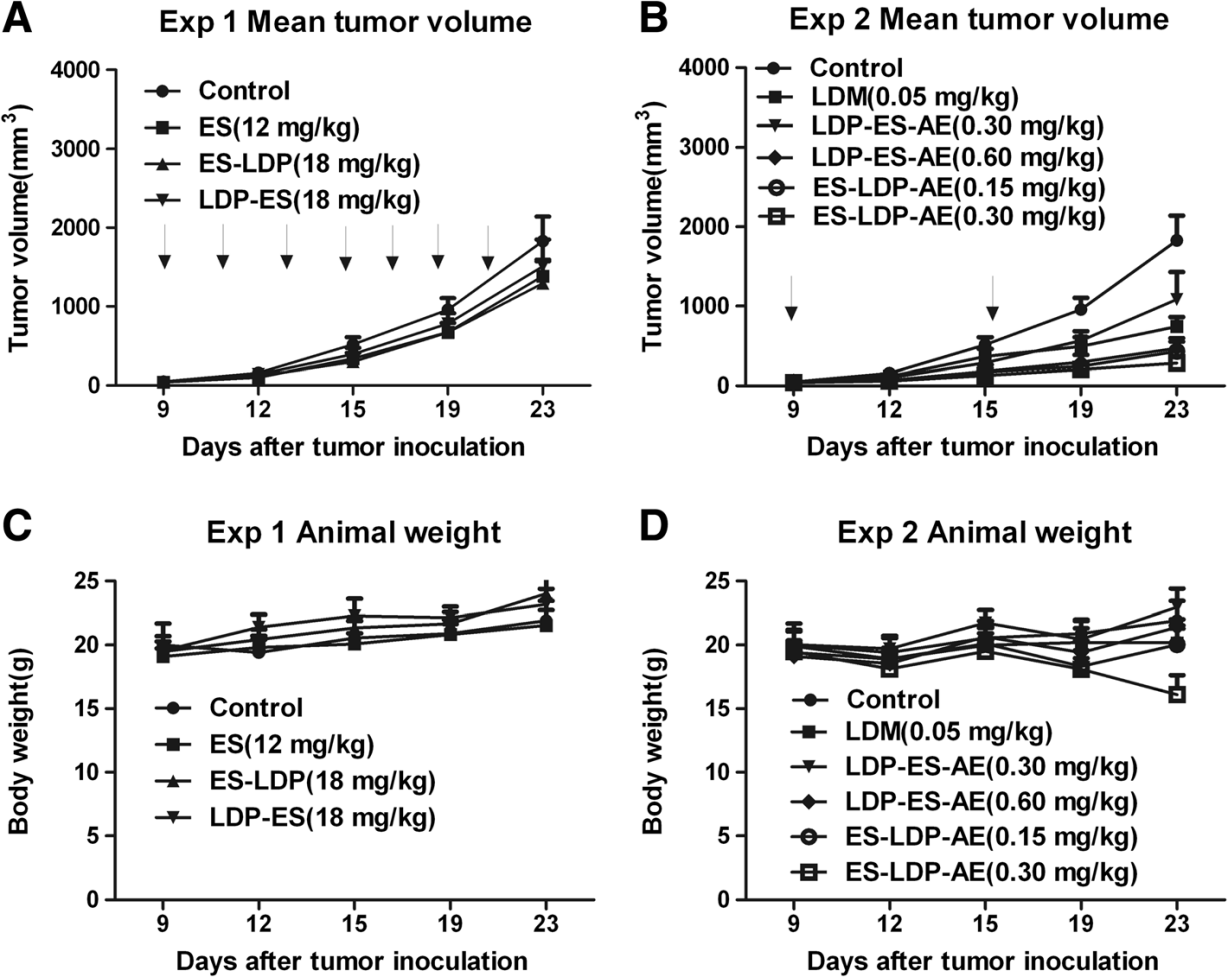
***In vivo *efficacies of ES**-**based fusion proteins and the enediyne**-**energized fusion proteins on PG**-**BE1 xenograft models.** In experiment 1 (Exp 1), nude mice bearing human lung carcinoma PG-BE1 xenografts were treated with ES, ES-LDP or LDP-ES i.p. injections at different doses (n = 6). Mean tumor volumes **(A)** and mean body weights of mice **(C)** in each group are shown. Arrows indicate the day of injection (every other day). In experiment 2 (Exp 2), nude mice bearing PG-BE1 xenografts were treated with LDM, LDP-ES-AE, or ES-LDP-AE (n = 6), i.v. injections respectively. Mean tumor volumes **(B)** and mean body weights of mice **(D)** in each group are shown. Arrows indicate the day of injection (day 9 and 15).

In the first experimental setting, the mice bearing PG-BE1 xenografts were divided into four groups and were treated with ES, ES-LDP, and LDP-ES, through intraperitoneal injection every other day in a total of 7 injections, respectively. ES was given at the dose of 12 mg/kg, and ES-LDP or LDP-ES was given at a dose of 18 mg/kg to meet the equal molar concentration with ES. Control mice received equal volume of saline. Determined by external measurement of tumor volume (twice per week), tumor growth was suppressed in ES and ES-LDP groups as compared with control mice over the whole period of 26 days. As evaluated on day 23, the inhibition rates of tumor growth for ES and ES-LDP were 24.5% and 30.2%, respectively, indicating moderate antitumor efficacies against the lung carcinoma PG-BE1 xenograft. By contrast, LDP-ES appeared to be less effective. Figure 6A shows the tumor growth curve of each group. Figure 6C shows the changes in body weight after treatment with ES and the fusion proteins, in which no significant difference was observed among the groups.

In a separate animal study, the antitumor activity of enediyne-energized fusion protein was investigated. PG-BE1 xenografts bearing mice were treated with LDM, ES-LDP-AE or LDP-ES-AE, respectively. LDM was given at a well-tolerated dose of 0.05 mg/kg. Because of the different enediyne assembly efficiency, ES-LDP-AE was given at the doses of 0.15 and 0.30 mg/kg; whereas LDP-ES-AE was given at the doses of 0.30 and 0.60 mg/kg, respectively. Mice received intravenous injection of LDM, ES-LDP-AE or LDP-ES-AE once a week for twice, and tumor volumes were measured during the treatment. It was shown that both energized fusion proteins have remarkable inhibitory effect on the growth of PG-BE1 xenografts (Figure 6B). Mice receiving LDM at 0.05 mg/kg showed an inhibition rate of 61.1%, while ES-LDP-AE (0.15 mg/kg) and LDP-ES-AE (0.60 mg/kg) at equivalent enediyne (AE) doses suppressed the tumor growth by 78.5% and 75.8% (P < 0.01, *vs* LDM), respectively (Figure 6B). Furthermore, the ES-LDP-AE-treated group at 0.30 mg/kg, inhibited tumor growth by 86.4%, showing significant difference (P < 0.01, *vs* LDM) compared with LDM-treated group at 0.05 mg/kg tolerated dose. Body weight loss resulted from the enediyne-energized fusion proteins treatment in each group was also measured at the termination of the experiment, in which all groups except ES-LDP-AE-treated group at 0.30 mg/kg did not exceed 10% of the pretreatment weights. No deaths were found in all treated groups (Figure 6D).

### ES-LDP inhibited tumor metastasis

**Figure 7:**
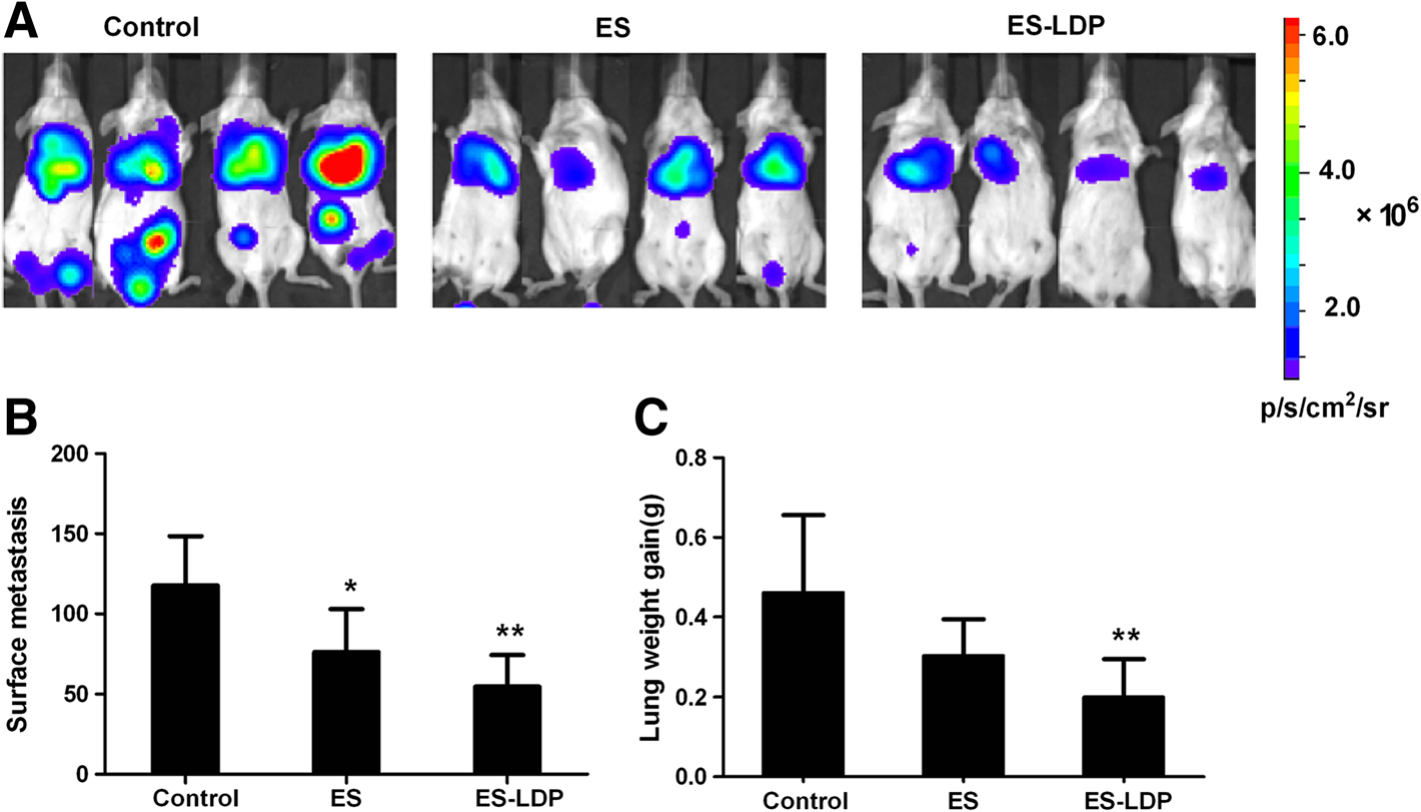
**ES**-**LDP treatment inhibits the lung metastasis of 4T1**-**luc tumors.** 4T1-luc cells (2 × 10^5^) were injected intravenously into mice. Mice were given ES (36 mg/kg body weight) or ES-LDP (54 mg/kg body weight) twice, and saline served as control. At the end of the experiment, mice were injected with D-luciferin and bioluminescence imaging was done. Then, animals were killed and lungs were harvested and weighed before evaluation of the surface metastasis. **(A)** The profile of optical imaging from different experimental groups. ES-LDP-treated mice showed both significantly reduced surface metastasis **(B)** and lung weight gain **(C)** in comparison to the control animals. (* P < 0.05,** P < 0.01; compared to control, respectively).

Because ES-LDP fusion protein was found to markedly suppress the migration of 4T1 cells *in vitro*, the anti-metastatic effect of ES-LDP was further evaluated with the lung metastasis model of 4T1-luc tumors. ES-LDP was administered through *i*.*v*. injections. Significant differences in lung colonization were found among the ES-treated group, ES-LDP-treated group and the untreated control group (Figure 7A). In addition, ES-LDP treatment significantly reduced the number of surface metastasis (51.4%) and lung weight gain (56.7%) in tumor-bearing animals compared to the untreated animals (Figure 7B and 7C). By contrast, ES decreased the number of surface metastasis and lung weight gain only by 35.2% and 34.2%, respectively.

## Discussion

ES, an angiogenesis inhibitor having been tested in multiple clinical trials, selectively targets endothelial cells in neovascularization and suppresses tumor growth. However, like other angiogenesis inhibitors, such as bevacizumab, sunitinib and sorafenib, ES could help patients to survive longer when given in combination with chemotherapy, but not when given alone [26]. To enhance the therapeutic efficacy of ES, several ES derivatives with different modifications have been designed, which include ES-cytosine deaminase protein, prolactin antagonist-ES, anti-HER2 IgG3-ES, ZBP-ES (Endostar), Fc-ES, and cell-permeable ES protein (HM_73_ES) [27-32].

The ES-cytosine deaminase protein, which converts a non-cytotoxic prodrug 5-fluorocytosine (5-FC) to the cytotoxic antitumor drug 5-fluorouracil (5-FU) in the local tumor area, significantly inhibited the growth of endothelial cells and preferentially induced tumor cell apoptosis [28]. The prolactin antagonist-ES fusion protein is a bifunctional protein, which inhibits both breast cancer cell proliferation and endothelial cell proliferation, exhibiting greater tumor inhibitory effects than prolactin antagonist and ES treated individually or in combination [29]. Targeting of ES using anti-HER2 antibody and human ES fusion protein could improve antitumor activity of either anti-HER2 antibody and/or ES and provides the versatile approach that could be applied to other tumor targets with alternative antibody specificities [30]. ZBP-ES, engineered by adding 9 extra amino acid residues MGGSHHHHH to the N-terminus of ES, showed increased thermodynamic stability and biological activity than the wild type ES [27], and was approved as anti-cancer drug in China. As reported, Fc-ES is a superior molecule to the original clinical ES. Due to its long half-life, the amount of protein required is substantially reduced compared with the clinically tested ES [31]. HM_73_ES exhibited enhanced tissue penetration and suppressed the growth of human tumor xenografts to a significantly greater extent than unmodified ES by adding a macromolecule transduction domain (MTD). Those results suggest another important mechanism to explain the enhanced activity of ZBP-ES and ES lacking the MTD sequence [32].

Recent studies indicated that LDP itself, the apoprotein of LDM, shows binding capability to a spectrum of human tissues, and notably that the binding capability correlates with the overexpression of EGFR and HER2 on the tumor tissue microarray [21]. In addition, LDP displayed moderate cytotoxicity to human hepatoma Bel-7402 cells with an IC50 value of 7.05 × 10^-5^ mol/l and it exerted tumor suppression on hepatoma H22 in Kunming mice [20]. The functional receptor of ES nucleolin was found to be specifically expressed on the surface of angiogenic blood vessels in tumor tissues, which endows ES low toxicity and tumor-specific distribution [7]. Therefore, in the present study, we constructed and prepared two ES-based fusion proteins ES-LDP and LDP-ES. Our results indicate that ES-LDP and LDP-ES disrupted the formation of endothelial tubule structures with the potency similar to that of ES. In addition, ES-based fusion proteins, especially ES-LDP, demonstrated much stronger inhibition of HMEC migration than ES. Furthermore, ES-LDP displayed high efficacy in PG-BE1 xenografts. This may be explained by the reason that N-terminal loop of ES around the zinc-binding site was involved in activity [33], and that the N-terminal integrity is essential for the biological functions of ES [27]. So it appears that the anti-tumor activity of ES could be enhanced by integrating with LDP and a free N-terminus of ES in the fusion protein is preferred. But its mechanism should be studied further, and there is more work to be done.

On the other hand, angiogenesis is involved in the development of distant metastasis. Thus by targeting angiogenesis, ES directly suppresses not only the growth of primary tumors but also metastasis. The present study has shown that ES-LDP or LDP-ES could markedly inhibit 4T1 cells migration in wound healing assay, with ES-LDP exhibiting a more potent activity. Therefore, we examined the effects of ES-LDP on the lung metastasis of 4T1-luc tumors. ES-LDP treatment significantly reduced the number of lung surface metastasis and lung weight gain in tumor-bearing animals. Therefore, ES-LDP could be explored as a novel therapeutic molecule in controlling metastasis of cancer.

LDM is considered as a highly potent “warhead” molecule for the construction of antibody-based tumor targeting drugs. At present, several LDM-containing energized fusion proteins have been manufactured with the two-step procedure in our laboratory, such as bispecific enediyne-energized fusion protein (Ec-LDP-Hr-AE) and tandem scFv-based enediyne-energized fusion protein (dFv-LDP-AE) [25,34]. Both of them possessed highly potent cytotoxicity to cancer cells and significant inhibitory efficacy *in vivo*. Ec-LDP-Hr-AE was more potent and selective in its cytotoxicity against different carcinoma cell lines *in vitro* and significantly inhibited the growth of SK-OV-3 xenografts in nude mouse model [34]. dFv-LDP-AE displayed extremely potent cytotoxicity to kinds of cancer cells, especially the lung cancer cell lines, and greatly increased the antitumor efficacy with lung carcinoma PG-BE1 xenograft in nude mice [25].

We adopted a strategy to energize the ES-based fusion proteins, ES-LDP and LDP-ES, with LDM enediyne chromophore to prepare ES-based and enediyne-energized fusion proteins (ES-LDP-AE and LDP-ES-AE). They all displayed potent antitumor activities against a variety of tumor cell lines with IC50 values ranged from 10^-9^ M to 10^-10^ M. Though the IC50 values had ten-fold difference, the IC50 value of ES-LDP-AE was always less than that of LDP-ES-AE. This difference may be due to the assembling efficiency of ES-LDP and LDP-ES, which was 83.9% and 27.1%, respectively. These results accord with assembling efficiency, and potential conformational change of the AE binding sites caused by the fusion. In the *in vivo* study, mice received tolerated dose of LDM at 0.05 mg/kg showed an inhibition rate of 61.1%. By contrast, ES-LDP-AE and LDP-ES-AE at equivalent doses suppressed the tumor growth by 78.5% and 75.8%, respectively. Furthermore, the ES-LDP-AE-treated group at higher dosage of 0.30 mg/kg showed an inhibition rate of 86.4%. No deaths were found in all treatment groups. As previously mentioned, ES has a unique ability for targeting therapy of cancer [15]. Endostar is now in clinical use for lung cancers in China, so we investigated the affinity of these ES-based fusion proteins to human lung cancers by tissue-microarray analysis. As shown, the positive percentage of ES and ES-LDP was higher than that of LDP; in addition, ES and ES-LDP share similar binding capability to lung cancer tissue, indicating that the fusion protein ES-LDP retains this capability as of the ES. It is of interest that the integration of LDP into the fusion protein ES-LDP does not compromise ES binding capability, while probably provides a targeting delivery of lidamycin.

## Conclusions

The ES-based fusion protein therapy provides some fundamental information for further drug development. Endostatin-lidamycin (ES-LDM) fusion proteins upon energizing with enediyne chromophore obtain the combined capability targeting tumor vasculature and tumor cell by respective ES and LDM moiety. Targeting both tumor vasculature and tumor cells by endostatin-based fusion proteins and their enediyne-energized analogs probably provides a promising cancer therapy.

## Abbreviations

AE: Active enediyne chromophore; ECM: Extracellular matrix; ES: Endostatin; IOD: Integrated optical density; LDM: Lidamycin; LDP: Lidamycin apo-protein; VEGF: Vascular endothelial growth factor.

## Competing interests

The authors declare that they have no competing interests.

## Authors’ contributions

WGJ carried out the cell experiments and was responsible for data analyses, manuscript preparation and editing. XAL constructed the vectors and DFZ helped to provide the fusion proteins. LL helped to prepare the enediyne-energized fusion protein. YL participated in IHC stainings from the TMAs. BYS and SHZ were involved in vivo study. YF helped with the interpretation of the results and with drafting the manuscript. YZL and YSZ designed the overall study, coordinated the study and helped to draft and finalize the manuscript. All authors read and approved the final manuscript.

## Pre-publication history

The pre-publication history for this paper can be accessed here:

http://www.biomedcentral.com/1471-2407/13/479/prepub
